# Forensic age estimation at the University Center of Legal Medicine Lausanne-Geneva: a retrospective study over 12 years

**DOI:** 10.1007/s00414-024-03254-8

**Published:** 2024-05-14

**Authors:** Frederique Thicot, Coraline Egger, Claudia Castiglioni, Virginie Magnin, Sana Boudabbous, Nikolaos Angelakopoulos, Silke Grabherr, Pia Genet

**Affiliations:** 1 Unit of Forensic Medicine, University Center of Legal Medicine Lausanne-Geneva (CURML), Geneva University Hospitals and University of Geneva, Rue Michel-Servet 1, 1211 Geneva 4, Switzerland; 2 Unit of Forensic Medicine, University Centre of Legal Medicine Lausanne-Geneva, Lausanne University Hospital and University of Lausanne, Chemin de La Vulliette 4, 1000 Lausanne 25, Switzerland; 3 Radiology Division, Diagnostic Department, Geneva University Hospitals (HUG), Geneva, Switzerland; 4 Department of Orthodontics and Dentofacial Orthopedics, University of Bern, Bern, Switzerland

**Keywords:** Forensic age estimation, Unaccompanied minors, AGFAD, Dental age, Bone age, Skeletal maturation

## Abstract

With the undeniable increase in asylum requests from unaccompanied alleged minors, age estimation of living individuals has become an essential part of the routine work in European forensic centers. This study aims to review the forensic age estimations performed in our center since 2010, to evaluate the state-of-the-art of this practice in Switzerland with the evolution of the methodology according to upcoming recommendations. Our institute's expert reports performed between 2010 and 2022 were retrospectively analyzed. We gathered the following parameters: demographic data, morphological characteristics, alleged age compared with the assessed minimum age, sexual maturation, dental and bone age. When available, we collected personal and family history, medical history, records of torture-related/self-inflicted injuries, and information about eating habits that might affect skeletal development. Data collection amounted to 656 cases. Forensic age estimations ordered by the Swiss Secretariat for Migration (SEM) represented 76.4% of cases, with 23.6% of them ordered by the Court/Public Prosecutor. Most alleged minors were male (94.5%) and came from Afghanistan (53.4%). Adjunction of CT scans of the sternoclavicular joints was necessary in 86.4% of cases. Only 25.2% of our reports concluded on most probable minority, with 55.6% of definite majors; in 19.2% of our cases, minority could not be excluded. This study aspires to further broaden our expertise regarding forensic age estimations. Given the increasing migratory flows, we can expect a notable increase in the frequency of these requests. Consequently, this study aims to promote a multidisciplinary approach and the international standardization of the methodology of these estimations.

## Introduction

Partly attributable to armed conflicts and socioeconomic challenges, cross-border migration has unquestionably surged in Europe in recent years. In 2022, Switzerland specifically recorded 2,450 asylum requests from unaccompanied minors, contrasting with the 989 requests documented in 2021 [1]. In instances where the minority status of an individual is uncertain, authorities may seek a forensic evaluation of the individual's age [2]. Forensic age assessments may be mandated by the Juvenile Court or the Public Prosecutor under circumstances where an individual, ostensibly a minor lacking a residence permit on Swiss territory, is placed on provisional detention in a prison for minors and the judge/prosecutor raises uncertainties about the individual's claimed minor status [3]. Additionally, the Swiss Secretariat for Migration (SEM) may initiate such assessments when an individual enters Swiss territory without valid or credible identification documents. The competence to conduct such assessments resides within forensic institutes, given that forensic pathologists possess specialized expertise in this domain. In recent years, the increase in migration inevitably led to a significant increase in the demand for forensic age estimations either ordered by the Court, the Public Prosecutor or the SEM. As in most European countries, the legal age of majority in Switzerland is fixed 18 years [4]. Nevertheless, in contrast to our neighbouring countries, the relevant age threshold establishing legal responsibility for an individual is set at 10 years old (art. 3 al. 1 DPMin) [5]. The age of 15 years also bears importance in the context of custodial sentences. Forensic age estimations find application in various domains such as civil law, competitive sports, and the identification of skeletal remains, etc. Ideally, the conduct of forensic age estimations should adhere to the recommendations set forth by the Study Group on Forensic Age Diagnostics of the German Society of Forensic Medicine (AGFAD) [6, 7] last updated in 2008. In Switzerland, guidance from the Swiss Society of Legal Medicine (SSML) [8], last revised in June 2022, is also integrated. These recommendations advocate for a comprehensive approach involving three pillar examinations, including an anamnesis and physical examination (to exclude developmental disorders or illnesses that could influence the sexual and the bone development), a dental examination (X-ray examination of the dentition, dental status) [9–13], and an evaluation of the skeletal development by examining an X-ray of the left hand/wrist (relying on Greulich and Pyle’s atlas) [14], and in case of complete maturation of the hand/wrist skeleton, a computed tomography (CT) scan of the sternoclavicular joints [6, 8] (with a recommended slice thickness ≤ 1 mm) [8]. Each pillar examination needs to be performed and analyzed by a specialist, with training and experience in forensic age estimations. A forensic pathologist conducts a comprehensive summary, interpretation of all the results, and draws conclusions. This professional undergoes annual proficiency tests organized by the AGFAD to ensure the optimal and continuous quality of these reports. However, adherence to these recommendations is not universal across all European medico-legal centers, resulting in disparities concerning the methodology and conclusions of forensic age assessment reports, as indicated by retrospective studies in the forensic literature [15–21]. As far as our knowledge extends, there has been no published analysis of forensic age estimations conducted on Swiss territory within the forensic literature. This study sought to assess the data pertaining to forensic age estimations conducted in our center throughout the last years. Additionally, this study aimed to evaluate the evolution of the methodology employed and the subsequent conclusions derived from these reports. The aim of this study was to assess the current state-of-the-art in forensic age estimations, to analyze the population undergoing age estimations in Switzerland and also to compare our results with other countries using different approaches.

## Material and methods

### Study population

We retrieved and scrutinized all forensic age estimation reports for the period spanning from 2010 to 2022 generated by the University Center of Legal Medicine Lausanne-Geneva (CURML), the institute in charge of forensic age estimations in the French and Italian speaking part of Switzerland (Latin) for the selected period, encompassing its three primary operational sites (Geneva, Vaud, Ticino). This region covers six cantons of the Swiss confederation, adding up to more than 2 million inhabitants (out of approximately 8.9 million inhabitants). The total study population for this research project comprised 663 forensic age assessment reports. These assessments were initiated either through court orders or by the SEM between 2010 and 2022. To perform the study, we systematically reassessed the forensic age estimation reports, along with the radiological reports and odontological reports. The inclusion criteria necessitated the execution of a minimum of two recommended pillar examinations by the AGFAD, with comprehensive reports from each examination category (anamnesis and clinical examination, bone age examination, dental age examination) being accessible. The exclusion criteria encompassed instances where more than one of the recommended AGFAD pillar examinations could not be conducted and/or the existence of documentation confirming a refusal for research.

### Data extraction

**Fig. 1:**
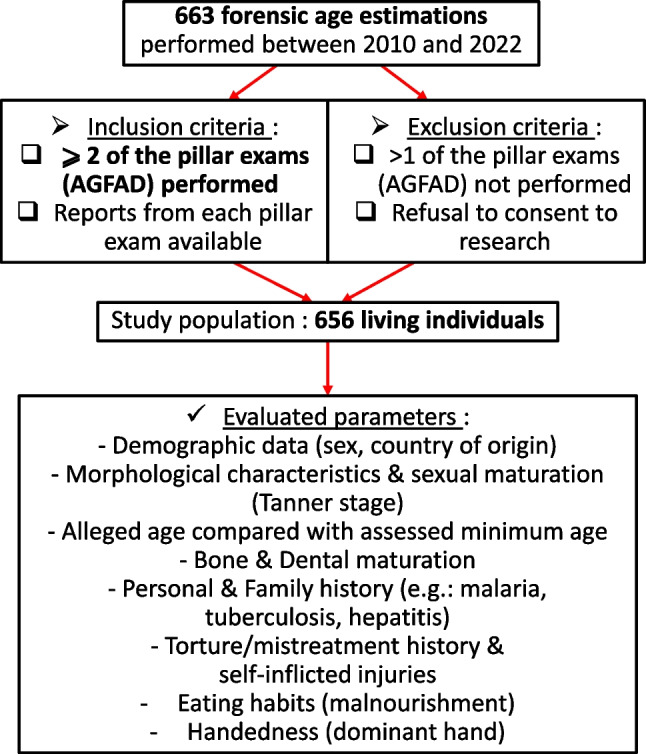
Flowchart showing the selection of the cases with inclusion and exclusion criteria and the evaluated parameters

All forensic age estimation reports pertaining to these 663 individuals were retrieved and reviewed to collect data on the following parameters: demographic information, morphological characteristics, alleged age compared to the assessed minimum age, sexual maturation (Tanner stage), dental and bone age. Additionally, when available, information regarding personal and family history, medical history, records of torture-related or self-inflicted injuries, handedness (dominant hand), and details about eating habits that could potentially impact skeletal development was gathered (Fig. 1). The alleged age of each subject was calculated by subtracting the alleged date of birth from the individual’s examination date. If individuals provided multiple birth dates, the date of birth corresponding to the youngest age was taken into consideration. The development of the hand skeleton was assessed based on the Greulich and Pyle atlas [14, 22] (occasionally coupled with the Thiemann-Nitz atlas [23]) and the dental status was based on the examination of third molar eruption (Olze et al. [24–26]) and mineralization according to Demirjian’s stages [9–13]. Results were then given based on reference studies with comparable ethnic groups [10, 12, 24, 25, 27–31]. Also, the assessment of the ossification of the clavicular medial epiphysis on CT scans was performed according to stage classifications by Schmeling et al. [32], adapted according to Kellinghaus et al. [33, 34] and Wittschieber et al. [35]. As supported by several studies, subjects were deemed to have reached majority when a stage 3c or more was obtained [36, 37]. According to the recommendations issued by the AGFAD, we applied the “minimum age principle” [6, 8]. The minimum age was determined according to the highest minimum age among those provided by the reference studies for the assessment of each developmental system [8].

### Statistical analysis

This research project primarily constitutes an exploratory study, with the objective of assessing the progression of our methodology and resultant conclusions over the years. This evaluation is conducted in alignment with the adjustments made in response to the forthcoming recommendations from AGFAD and SSML. General descriptive statistics and further hypothesis tests were performed in search of a potential correlation, using IBM’s Statistical Package for the Social Sciences (SPSS) version 28. The threshold of significance was set at 5%. The study was conducted with the approval of the biobank of our institute, the local ethics board (registration number 2023–01575) and the SEM. Given the retrospective nature of the study and the utilization of anonymized patient data, the ethics board waived the need for informed consent. The study was carried out in accordance with the ethical standards laid down by the Declaration of Helsinki (Finland) [38].

## Results

### Population data

**Fig. 2:**
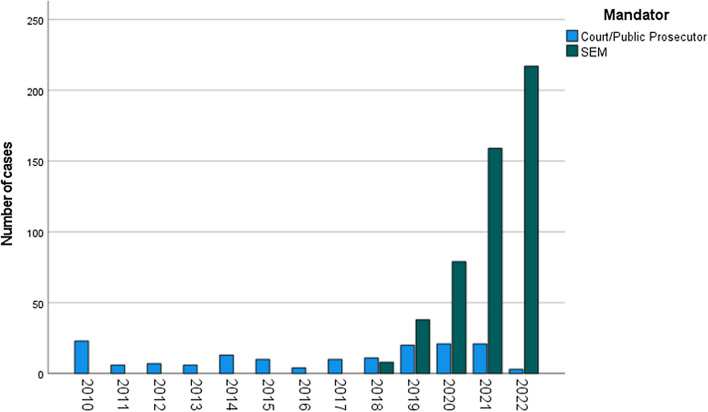
Graph: Mandator’s distribution over the years; blue: age estimations demanded by the Court/Public Prosecutor; green = age estimations demanded by the SEM

**Fig. 3:**
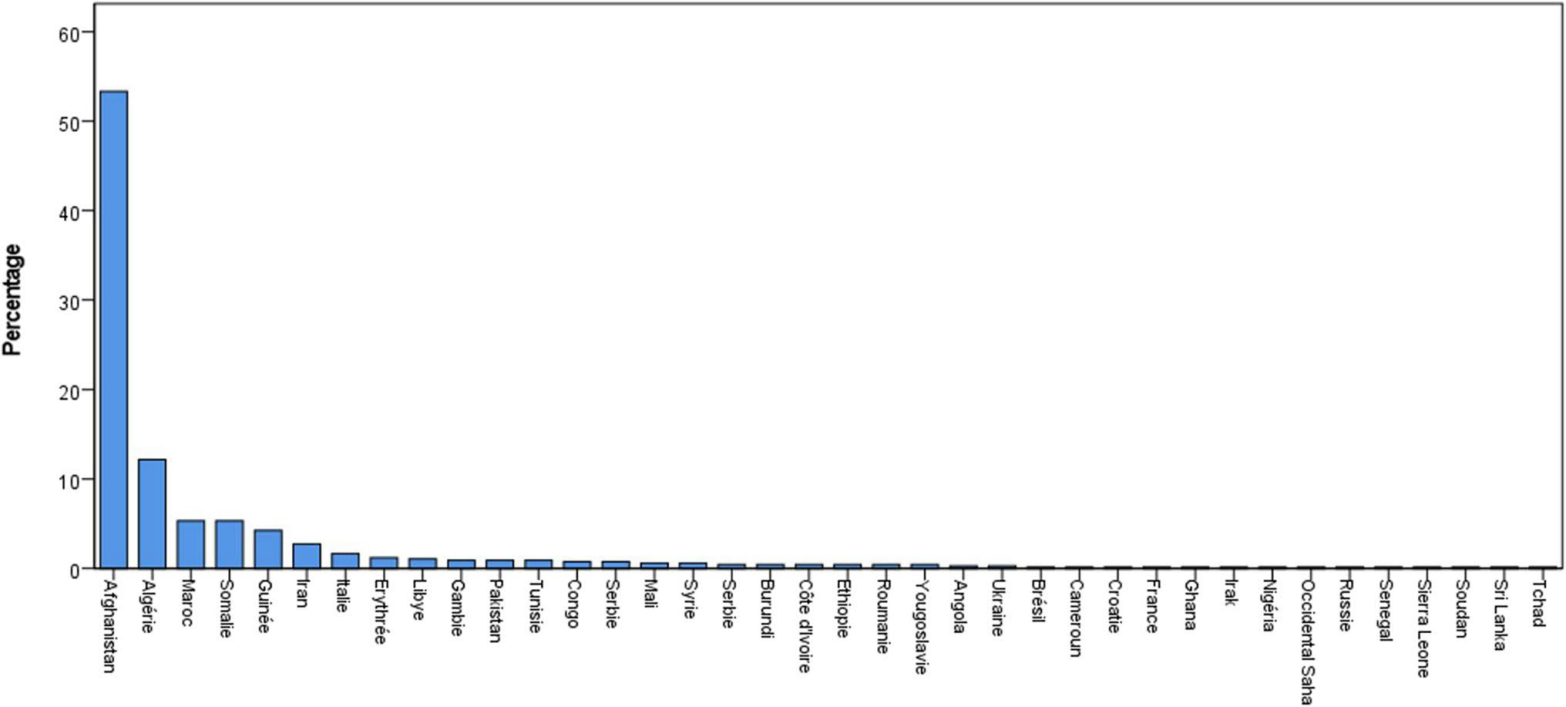
Graph: Demographic distribution of the study population with indication of the country of origin

According to the application of the inclusion and exclusion criteria, the final study cohort comprised 656 individuals. At first glance, we observed that 76.4% of the forensic age assessments performed were at the behest of the SEM (501 cases vs 155 cases). A comprehensive breakdown of the case distribution across the years is presented in a bar graph (Fig. 2). Looking into the population itself, our sample was mainly composed of males (94.5%, *n* = 620). Most alleged minors came from Afghanistan (53.4%), Algeria (12.2%), Morocco and Somalia (5.3% each) followed by Guinea (4.3%). All demographics are detailed in Fig. 3.

### Descriptive analysis

We exclusively utilized the X-ray modality in 13.6% (*n* = 89) of cases, while adhering to the AGFAD recommendations necessitated the incorporation of a CT scan of the sternoclavicular joints in 86.4% of cases (*n* = 567). However, it is worth mentioning that the sternoclavicular CT scan was not a component of the standard protocol during the initial years of our study period. More precisely, 86.5% of males (*n* = 536) and 66.7% of females (*n* = 24) showed complete maturation of the hand/wrist skeleton (respectively standard 31 and 27 of the Greulich and Pyle’s atlas). According to the classification of the ossification status of the medial clavicular epiphysis [33, 34], the most represented stages were 3c and 4 (respectively 44.7% (*n* = 243) and 14% (*n* = 76) within males and 21.7% each within females (stage 3c, *n* = 5, stage 4, *n* = 5), therefore implying majority (minimum age of 19 years old in males and 19.4 in females for the 3c stage). Furthermore, 61.9% of our study population subjected to a CT scan (*n* = 351) showed an ossification stage corresponding to stages 3c to 5. Notably, a small percentage of cases showed an irregular or unclassified epiphyseal ossification, unilaterally in approximately 9% of cases (*n* = 52 (left side) and *n* = 53 (right side)) and bilaterally in 6.7% of cases (*n* = 38) [39]. On the other hand, examination of third molars showed complete development (Demirjian stage H) in 66.7% (*n* = 430), followed by stages G (15%, *n* = 97) and F (8.7%, *n* = 56). It is noteworthy to mention that 4.3% of subjects (*n* = 28) did not have third molars.

### Comparative results

**Fig. 4:**
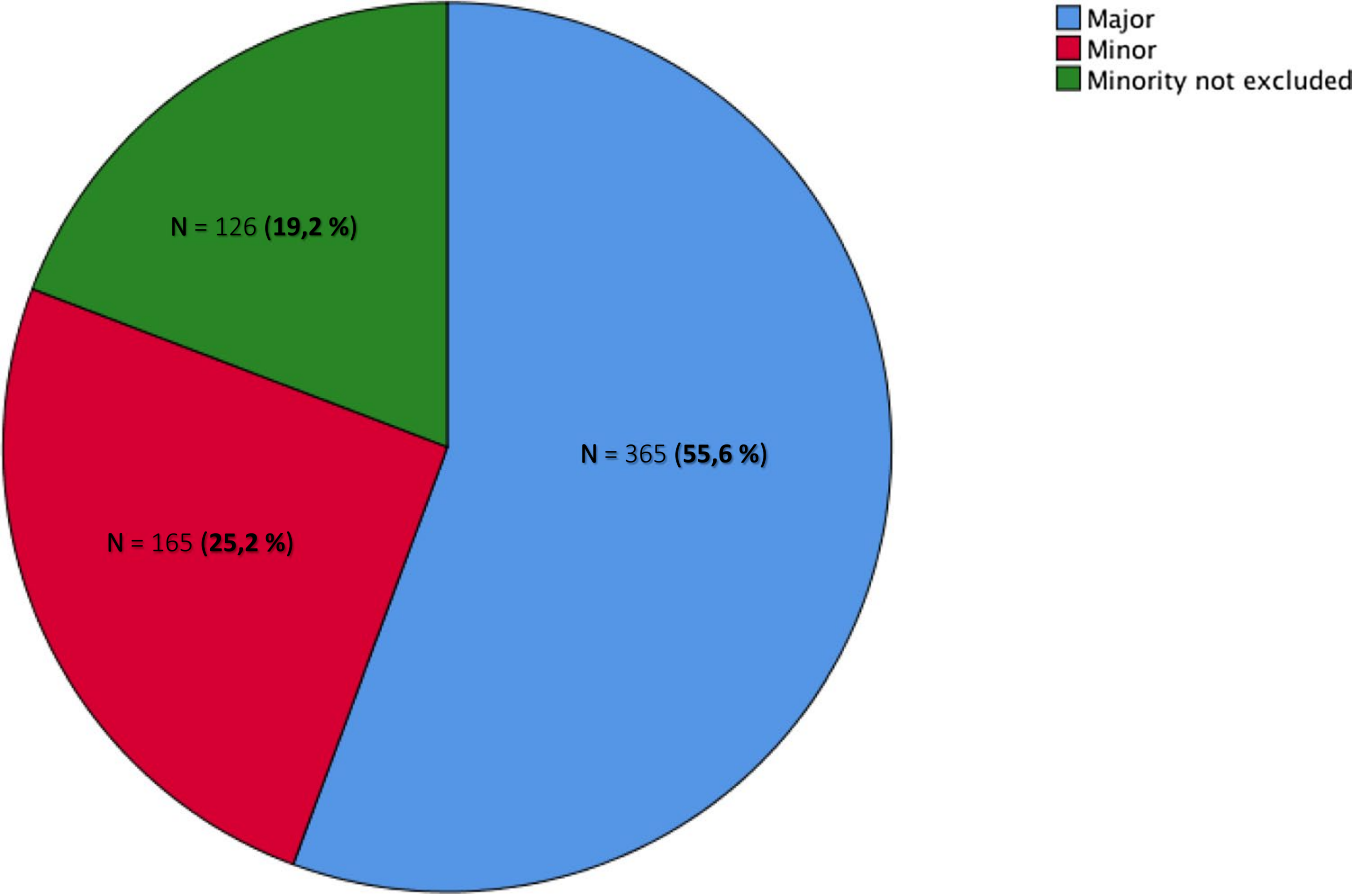
Diagram: Report’s conclusions according to majority (blue), minority (red), border line cases where minority couldn’t be excluded (green)

We detected a significant difference (ranging from 3 to 10.2 years) between the mean dental and bone age in 7.9% of cases (*n* = 52), with bone age predominantly exceeding the dental age (71.2%, *n* = 37). Indeed, dental development is considerably less influenced by endocrine disorders than bone maturation [40]. On average, these minors alleged a mean age of 16.664 years old (Std dev.: 1.5441, range: 11.1–43.6), while we assessed a mean minimum age of 18.205 (Std dev.: 2.123, range: 12.1—26.0). As illustrated in Fig. 4, our reports concluded that the individual was major in 55.6% (*n* = 365), and minor in 25.2% (*n* = 165) of the cases. However, we could not eliminate the possibility that the individual was a minor in 19.2% of cases (*n* = 126), mainly when a stage 3b of the sternoclavicular joints was observed on the CT scan (corresponding to a minimum age of 17.6 years). Moreover, we could exclude the alleged age in 77.3% of cases (*n* = 507).

### Interfering diseases and personal history

In terms of medical history, we retrieved 16 individuals (2.4%) who reported to have suffered from malaria. One of these male individuals showed a significant difference between dental age (Demirjian H, lowest mean value: 18.5 years old according to the evaluating dentist) and bone age (Greulich/Pyle standard 29, minimum 14.9 years old). More interestingly, another male case showed a complete dental development (Demirjian H, lowest mean value: 20.7 years old according to the evaluating dentist) with incomplete ossification of the medial clavicular epiphysis (CT stage 2a, minimum 14.4 years old (case of 2013)). We also identified 8 cases of past tuberculosis (1.1%), 5 cases of hepatitis B infections (0.9%) and only one case of thyroid disorder. Nevertheless, none of these individuals exhibited a substantial disparity between dental and bone age. Regarding personal history (when available), it was noted that 12.1% of subjects reported a history of torture (*n* = 45). Additionally, 71% claimed to have experienced mistreatment during their journey (*n* = 304), and 8.4% exhibited indications of self-inflicted injuries (*n* = 53). In addition, most of the subjects reported having a balanced diet (68.5%, *n* = 382). Malnutrition was mostly reported during the migratory journey. It is worth mentioning that 73.3% of the studied population (*n* = 481) refused the assessment of sexual maturation (genital/breast examination), probably due to modesty and religious reasons. However, 94.9% of the assessed subjects (*n* = 175) showed complete sexual maturation (Tanner stage 5) and no evidence of developmental disorder was mentioned.

### Intra-individual differences in the results of sternoclavicular CT scans

While gathering and reviewing all the CT data, we observed a small proportion of intraindividual differences in the maturation of the sternoclavicular joints. Indeed, out of the 567 CT scans, 15.1% (*n* = 99) showed different stages of ossification between the right and left side. However, those differences rarely exceeded two substages (e.g., 3a and 3c) except in one case exhibiting stages 2a on one side and 3a on the other. We then hypothesized that handedness (dominant hand) might influence the ossification levels of the medial epiphyseal plates of the clavicles on CT scans of the sternoclavicular joints. Among the 99 instances of different stages of maturation between the right and left side, information about handedness was available for only 71 (71.7%). In slightly more than half of these cases (*n* = 40, 56.3%), the dominant side exhibited a more advanced stage of ossification of the medial clavicular epiphysis, although the difference was not statistically significant (chi-square test: *p* = 0.412).

## Discussion

We reviewed 656 forensic age estimations performed in Switzerland between 2010 and 2022. As portrayed in our study, the SEM ordered three times more forensic age assessments than the Court or the public prosecutor, only since 2018, escalating to nearly 70 times more by 2022. In alignment with the numbers released by the SEM, most subjects were males (94.5% in our study versus 96.5% according to the SEM report from 2022 [1]) coming from Afghanistan (53.4% in our study versus 81.6% according to the SEM report from 2022 [1]). The demographic characteristics illustrated in our sample exhibited slight variations from recent data published by neighbouring countries such as France and Spain, with subjects predominantly coming from Guinea in a recent study published by Lossois et al. [15] (Montpellier), and from Morocco in another study by Taranilla Castro et al. [16] (Barcelona). In Italy [41], most unaccompanied minors came from Nigeria, Eritrea or Guinea. However, our results are quite similar to published data from Germany [18], Finland [19] and Austria [20]. On the other hand, existing literature on forensic age estimations consistently portrays a predominance of male subjects in the studied populations.

Within our cohort, most individuals (66.7%, *n* = 430) presented a complete development of at least one third molar (maxillary or mandibular) (Demirjian stage H) followed by a small subset of subjects (15%, *n* = 97) showing third molar roots near completion (Demirjian stage G). These results are in line with other studies published by neighbouring European countries [15, 16, 20]. In our study, a complete development of the skeleton of the hand was observed in the vast majority of cases (86.5% of males and 66.7% of females), necessitating the inclusion of a sternoclavicular CT scan. This compares with the published numbers by Lossois et al. [15] reporting 98% of complete ossification out of their 265 cases, whereas Taranilla Castro et al. [16] only observed 40.5% of complete ossification out of their 2,754 cases. Out of the 567 CT scans at our disposition, 61.9% of subjects (*n* = 351) showed a clavicular epiphyseal ossification stage corresponding to a stage 3c to 5. These results are quite similar to published data from Austria [20] and France [15]. We concluded that 25.2% of our subjects were definite minors, while in 19.2% of cases, minority status could not be excluded. These results differ to our French neighbours with approximately 4.15% of minors in Montpellier [15], as opposed to 71.2% of minors in Barcelona [16]. The variance in results can be explained by the divergence in standards, procedures, and legal requirements across medico-legal centers and countries in conducting age estimation assessments, and the absence of uniform adherence to AGFAD's recommendations. In Barcelona, for example, as elucidated by Taranilla Castro et al. [16], the practice of such examinations and expertise did not align with AGFAD's recommendations (at the time of the study); instead, it adheres to a Spanish consensus. Indeed, only 15 CT scans were performed out of the 2754 subjects, because they only performed a CT scan when there was no third molar accompanying complete ossification on X-ray [16]. As indicated by a recent survey conducted by the European Asylum Support Office, only 12 out of 30 European countries incorporate a radiological examination of the clavicles in the age assessment process for unaccompanied minors with uncertain age minority [18, 42]. It should be mentioned that the aforementioned study by Taranilla et al. [16] did not provide explicit details on the method or criteria employed for establishing a minimum age determination. Hence, while it is possible to compare the percentage of conclusions favoring age majority or minority, a direct comparison of the methods employed to reach these conclusions is not feasible.

Conversely, Lossois et al. [15] also applied the minimum age principle recommended by the AGFAD to determine the minimum age, reaching a conclusion favoring majority beyond reasonable doubt in a comparable proportion to our study (49.4%). However, because they determined a “most probable age”, they concluded that 95.85% of their entire cohort had most probably or unequivocally reached the age of majority [15]. Kreitner et al. [43] were the first to observe differences in development between the left and right medial clavicular epiphyses in 1.6% of their pilot trial applying thick-slice CT imaging. Further studies later chose to either select the more or the least advanced stage of ossification to estimate the age, while some chose to calculate the ossification stage for each side and summarize the results [44, 45]. However, those intra-individual differences do not seem to exceed one main stage [40, 44] and the lesser differentiated stage tended to be encountered alongside the dominant hand [40]. Our investigations regarding the possible origins of the difference in ossification of the clavicular medial epiphysis (such as handedness, and history of trauma/surgery) were inconclusive. In contrast to the findings presented by Schmeling et al. [40], our results revealed a more advanced stage of ossification in the medial clavicular epiphysis on the side of the dominant hand in 56.3% of cases. However, subsequent research with a larger sample size is imperative to properly test this hypothesis.

It is well known that certain diseases (e.g. tuberculosis, hepatitis and malaria) and a low socioeconomic status (especially low access to nutrition) can influence the development, especially the bone age, provoking a delay in growth and, therefore, inducing an underestimation of the actual age [8, 46, 47]. In our cohort, we identified a small subset of individuals with a past history of malaria, with only two subjects exhibiting a significant difference between dental and bone age, the latter being less advanced.

### Limitations and future directions of this research

Regrettably, we have not yet been able to establish a correlation between our estimations and the ultimate decisions rendered by the Administrative Federal Court (TAF). However, this represents a prospective objective for subsequent research. Nevertheless, this research holds the merit of being an inaugural retrospective review of forensic age estimations conducted on Swiss territory. This project has the potential to pave the way for future comparisons among various samples from different forensic institutes in Switzerland. It should also be mentioned that we have not come across other published studies on forensic age estimations that have explored torture-related or self-inflicted injuries, along with nutritional habits, as extensively as undertaken in this research.

Nevertheless, we identified several limitations in this study, including a lack of uniformity in the methodology of the reports over the years. Indeed, earlier reports did not comply with the later issued recommendations from the AGFAD (especially the adjunction of a CT scan and the minimum age principle), resulting in some unexpected results. As previously mentioned, the inclusion of the sternoclavicular CT scan was not part of the standard protocol during the initial years of our study period and was only fully implemented in 2012. Therefore, we were able to retrospectively identify 12 cases in which a CT scan of the sternoclavicular joints would have been indicated. As an example, one of our reports from 2011 concluded that the individual had reached the age of majority solely based on the complete ossification of the hand skeleton (Standard 31) despite the absence of third molars. It is worth mentioning that two protocol adjustments were performed in our forensic center in 2013 and 2021 according to upcoming recommendations. We could then observe an evolution and adaptation of our practice over the years, complying with the most recent recommendations. Another limitation is that the data were collected through a retrospective review, implying that certain relevant information may not have been accessible for every case. Moreover, variability inherent to the expert in forensic pathology signing the report regarding the amount of information collected (e.g., handedness only available in a handful of cases) and the extent of physical examination (e.g., sexual maturation assessment by examination of the genitalia) must be considered. This situation can also be explained by the necessity of treating each case individually, considering the specificities that are unique to each individual when drawing conclusions for each report. Ultimately, the modest sample size and heterogeneity among cases constrain the generalizability of these findings.

## Conclusion

This research allowed us to provide a detailed analysis of the population undergoing forensic age estimations in Latin Switzerland, sharing similarities with some neighbouring countries (male, Afghanistan). When comparing our results with other published research in Europe, significant differences arose in terms of conclusions of forensic age estimations, depending on the guidelines and standards applied. Forensic age assessment remains an estimation of age; a precise determination of age is not possible. However, the precision of these estimations is increased by the use of the “three pillar method” proposed by the AGFAD, which exhibits substantial internal coherence and correlation between dental and bone age, as evidenced in our review. It is essential to bear in mind the "in dubio pro minore" principle; from our perspective, prioritizing the minimum age principle is crucial. The discrepancies highlighted in this paper, and the comparison with other recent studies concerning various European countries, emphasize the imperative necessity for harmonization of the current practices in Europe. Subsequently, such harmonization on a global scale is crucial to ensure consistent treatment of alleged minors and to uphold uniform procedures and standards.

## Data Availability

Under request all anonymous data can be asked to the corresponding author.
